# Detection of herpes viruses in the cerebrospinal fluid of adults with suspected viral meningitis in Malawi

**DOI:** 10.1007/s15010-012-0292-z

**Published:** 2012-07-14

**Authors:** L. A. Benjamin, M. Kelly, D. Cohen, F. Neuhann, S. Galbraith, M. Mallewa, M. Hopkins, I. J. Hart, M. Guiver, D. G. Lalloo, R. S. Heyderman, T. Solomon

**Affiliations:** 1 Brain Infections Group, Walton Centre NHS Foundation Trust, and Institute of Infection and Global Health, University of Liverpool, The Apex Building, 8 West Derby Street, Liverpool, L69 7BE UK; 3 Liverpool Specialist Virology Centre, Royal Liverpool and Broadgreen University Hospitals NHS Trust, Liverpool, L7 8XP UK; 4 Institute of Public Health, Universitätsklinikum Heidelberg, Im Neuenheimer Feld 324, 69120 Heidelberg, Germany; 5 Department of Clinical Virology, Manchester Royal Infirmary, Manchester, M13 9WL UK; 6 Liverpool School of Tropical Medicine, Liverpool, L3 5QA UK; 7 Malawi–Liverpool–Wellcome Trust Major Overseas Programme, Blantyre, Malawi

**Keywords:** Adults, Africa, Central nervous system infections, Herpes virus, HIV, Viral meningitis

## Abstract

**Purpose:**

We looked for herpes simplex virus types 1 and 2 (HSV-1 and HSV-2, respectively), varicella zoster virus (VZV), Epstein–Barr virus (EBV) and cytomegalovirus (CMV) DNA in Malawian adults with clinically suspected meningitis.

**Methods:**

We collected cerebrospinal fluid (CSF) from consecutive adults admitted with clinically suspected meningitis to Queen Elizabeth Central Hospital (QECH), Blantyre, Malawi, for a period of 3 months. Those with proven bacterial or fungal meningitis were excluded. Real-time polymerase chain reaction (PCR) was performed on the CSF for HSV-1 and HSV-2, VZV, EBV and CMV DNA.

**Results:**

A total of 183 patients presented with clinically suspected meningitis. Of these, 59 (32 %) had proven meningitis (bacterial, tuberculous or cryptococcal), 39 (21 %) had normal CSF and 14 (8 %) had aseptic meningitis. For the latter group, a herpes virus was detected in 9 (64 %): 7 (50 %) had EBV and 2 (14 %) had CMV, all were human immunodeficiency virus (HIV)-positive. HSV-2 and VZV were not detected. Amongst those with a normal CSF, 8 (21 %) had a detectable herpes virus, of which 7 (88 %) were HIV-positive.

**Conclusions:**

The spectrum of causes of herpes viral meningitis in this African population is different to that in Western industrialised settings, with EBV being frequently detected in the CSF. The significance of this needs further investigation.

## Introduction

In African settings, aseptic meningitis is thought to be common, but the aetiology is rarely identified and there have been few studies examining this question [1, 2]. In industrialised countries where a more thorough diagnostic work-up is possible, up to 50 % of cases have been found to have a viral cause [3, 4].

Reactivation of herpes simplex virus (HSV) types 1 and 2, varicella zoster virus (VZV), Epstein–Barr virus (EBV) and cytomegalovirus (CMV) are common in the context of human immunodeficiency virus (HIV) infection and HIV–herpes virus co-infection has been linked with a more severe disease course [5, 6]. Whilst all five viruses can cause meningitis in HIV-infected adults, EBV infection is more usually associated with central nervous system (CNS) lymphoma, whilst CMV is more frequently the cause of encephalitis, radiculitis or retinitis [5, 7–9].

In sub-Saharan Africa, very little is known about the prevalence of HSV, VZV, EBV and CMV as causes of aseptic meningitis in the context of HIV. We, therefore, looked for HSV, VZV, EBV and CMV infections in Malawian adults with clinically suspected meningitis, but without evidence of bacterial or fungal infection.

## Methods

### Patients and CSF samples

This study was a sub-analysis of cerebrospinal fluid (CSF) from a cohort of adults (≥16 years of age) with clinically suspected meningitis admitted to Queen Elizabeth Central Hospital (QECH), Blantyre, Malawi, from September through to December 2007 [10]. Demographics and clinical information were collected using a standardised case record form. All persons gave their informed consent prior to inclusion in the study. Routine microscopy and culture of the CSF was performed and patients with microbiologically confirmed bacterial, cryptococcal or tuberculous meningitis were excluded, as were those with probable bacterial meningitis (CSF white cell polymorphs >100 cells per mm^3^). Patients with a diagnosis of a CNS infection within the last month were also excluded; this was primarily done to ensure that our cohort of patients had new clinical events and to avoid selecting those with a relapse of past CNS infection. Patients with ≥5 white cells per mm^3^ of CSF were defined as having an aseptic meningitis, whilst those with <5 white cells per mm^3^ were defined as not having meningitis using a laboratory diagnostic criteria [11]. A CD4 count of <200 cells/µl was used to define advanced HIV disease.

The study was approved by the local ethics committee at the College of Medicine, University of Malawi, Blantyre, Malawi (P05/06/471) and the ethics committee medical faculty, University Heidelberg, Heidelberg, Germany (S 026/2007).

### Herpes virus detection

CSF samples were aliquoted and stored at −70 °C prior to molecular diagnostics for HSV, VZV, EBV and CMV. The samples were tested as a batch at the time of this study.

DNA was extracted from 200 µl of CSF using a QIAamp DNA kit as per the manufacturer’s instructions. DNA recovery was confirmed by spectroscopic analysis. We used 5 µl of DNA for each polymerase chain reaction (PCR) for the HSV, VZV and EBV assays, and 10 µl for the CMV assay.

Primers and probes targeting conserved regions of the DNA polymerase gene were used for HSV-1 (primer: forward-GAC AGC GAA TTC GAG ATG CTG, reverse-ATG TTG TAC CCG GTC ACG AAC T; probe: 5′-FAM-CAT GAC CCT TGT GAA ACA-MGB-3′-BHQ1); HSV-2 (primer: same as HSV-1; probe: 5′-VIC-TGA CCT TCG TCA AGC AG-MGB-3′-BHQ1) and VZV (primer: forward-GCG CGG TAG TAA CAG AGA ATT TC, reverse-ACG TGC ATG GAC CGG TTA AT; probe: 5′-FAM-ACC ATG TCA TCG TTT CAA-MGB-3′-BHQ1). Similarly, the tegument protein (BNFR1 gene) for EBV and UL123/UL 55 genes for the CMV genome were targeted [12, 13].

Applied Biosystems (ABI, UK) AmpliTaq Gold (2×) Master Mix was used for all the real-time PCR assays. All reactions were carried out in the ABI 7300 real-time PCR machine using 96-optical-well plates, with a total volume of 25 µl per well. The PCR thermocycler conditions for all assays included three stages; activation of UNG at 50 °C for 2 min, activation of Taq at 95 °C for 15 min, followed by amplification using 45 cycles at 95 °C for 15 s and 60 °C for 1 min.

A positive control (provided by the National Institute for Biological Standards and Control, UK) for each herpes virus was run with unknown specimens to ensure DNA recovery. Similarly, specimens were processed in parallel with aliquots of molecular-grade water to monitor for false-positive results; the run was discarded if this was the case. Each individual sample was processed in triplicate and the sample was considered to be positive if two or more of the three test results were positive. When only one out of the three test results was positive, the PCR test was repeated (*n* = 3) and the sample was then considered to be positive if the test result was positive (*n* = 1). Quantification was only performed with EBV and shown as genome copies per millilitre of CSF. Serial dilution of 10^0^–10^8 ^copies/ml of virus stock was used to obtain a standard curve with an efficiency performance of 92 %. The concentration of EBV genome in the samples was interpolated from this standard curve.

VZV is known to have high genotype diversity [14]. To ensure that the PCR primers were appropriate for VZV strains circulating in Malawi, swabs (stored in viral medium) taken from the vesicles of four patients with classical shingles (vesicular rash in a dermatomal distribution) were also analysed. The vesicular samples of all four patients with shingles gave positive results.

A sensitivity analysis was performed to determine the limit of detection using QCMD panels (Qnostics Ltd., UK). We found the limit of detection to be 10^3^ copies/ml or less with each virus.

### Statistical methods

The data were analysed using PASW Statistics 18, Release Version 18.0.0 (Ó SPSS, Inc., 2009, Chicago, IL, http://www.spss.com). Quantitative variables were expressed as medians with interquartile ranges and qualitative variables as frequencies and percentages. Fisher’s exact test was used to compare categorical data and the Mann–Whitney *U* test for continuous data; a *p* value of <0.05 was taken to be statistically significant.

## Results

**Fig. 1:**
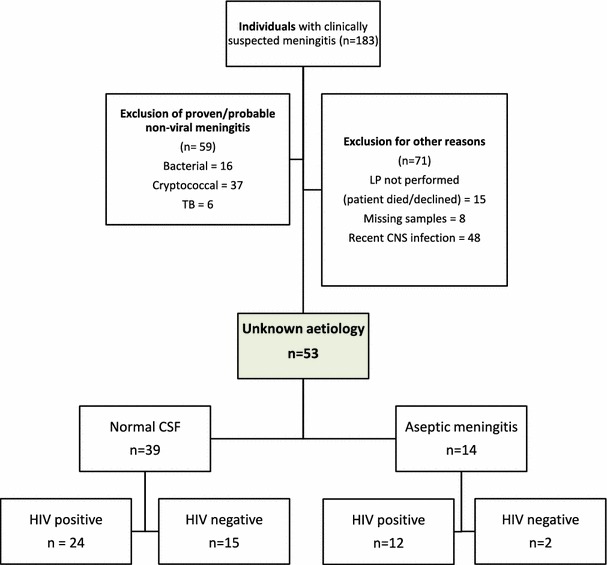
Flow diagram describing the selection of eligible participants and their classification. *CNS* central nervous system, *CSF* cerebrospinal fluid, *LP* lumbar puncture

A total of 183 patients presenting with clinically suspected meningitis were identified over the 3-month study period. Of these, 59 (32 %) had proven meningitis (bacterial, tuberculous or cryptococcal), 39 (21 %) had normal CSF and 14 (8 %) had an aseptic meningitis. In 48 (26 %) patients, there was a relapse of a previous CNS infection (Fig. 1). Thirty-six (68 %) of 53 patients with a normal or aseptic CSF were HIV-positive, with a median CD4 count of 159 mm^3^ (interquartile range 107–270 mm^3^).

Overall, in the aseptic meningitis group, a herpes virus was detected in the CSF of 9/14 (64 %); all of them were HIV-positive. Amongst those with a normal CSF, 8/39 (21 %) had a detectable herpes virus; of these, 7 (8 %) were HIV-positive.

The prevalence of the individual herpes virus in those with aseptic meningitis (14) was 7 (50 %) EBV and 2 (14 %) CMV. In those with a normal CSF (39), 6 (15 %) had EBV and 2 (5 %) had HSV-1 detected. No patient had HSV-2 or VZV detected (Table 1).

**Table 1:** Demographics, CSF characteristics and detection of herpes viruses in 53 patients with suspected viral meningitis

Aetiology	Patients with virus detected in CSF, no. (%) (*n* = 53)	Males, no. (%)	Age, median (IQR)	HIV-positive, no. (%)	CSF WCC, cells/mm^3^, median (IQR)	CSF protein mg/dl, median (IQR)
HSV-1	2 (4)	2 (100)	35.5 (30, 41)	1 (50)	0 (0, 0)	36 (25, 52)
HSV-2	0	–	–	–	–	–
VZV	0	**–**	**–**	–	**–**	**–**
EBV	13 (25)	4 (31)	35 (28, 42)	13 (100)	17 (0, 310)	68 (46, 203)
CMV	2 (4)	0	35 (30, 40)	2 (100)	2140 (200, 4,080)	403 (278, 528)
No virus detected	36 (68)	15 (42)	35 (25, 40)	21 (58)	0 (0, 0)	36 (25, 52)
Any virus detected	17 (32)	6 (35)	33.5 (26, 42)	16 (94)	0 (0, 23)	50 (29, 72)

*IQR* interquartile range, *WCC* white cell count, *CSF* cerebrospinal fluid

When comparing the rate of detection in the HIV-positive and HIV-negative patients, we found that only one HIV-negative patient had a herpes virus detected in the CSF (HSV-1), compared with 16 HIV-positive patients. Thirteen (36 %) patients with HIV infection had EBV detected in the CSF.

To investigate the relevance of the high EBV prevalence found exclusively in the HIV-positive group, we explored the relationship between CSF EBV titre, CSF white cell count (WCC) and CD4 count. The median titre of EBV in those with a normal CSF (<5 cells/mm^3^) was 1,202 copies/ml, compared to 6,680 copies/ml in those with an abnormal CSF (≥5 cells/mm^3^) (*p* = 0.048). The median CSF EBV titre in patients with advanced HIV infection was 2,223 copies/ml, compared with 1,239 copies/ml in those with less advanced disease (*p* = 0.52).

## Discussion

We have found that aseptic meningitis constituted 8 % of the patients with clinically suspected meningitis in our setting. Surprisingly, no HSV-2 or VZV DNA was detected in the aseptic CSF; instead, we detected a high prevalence of EBV and all of these patients were HIV-positive.

The prevalence of aseptic meningitis in our cohort was lower than in Western industrialised countries, where aseptic meningitis accounts for about 50 % of acute meningitis [15]. As far as we know, there are only two other publications which investigated aseptic meningitis in sub-Saharan Africa; one in Zimbabwe found the frequency of mononuclear meningitis to be 27 %, whilst the other in South Africa found the frequency to be 59 %; neither searched for viral aetiology [1, 2].

It is conceivable that the low frequency could be due to an ascertainment bias, as patients with aseptic meningitis have milder self-limiting disease and, therefore, do not present to hospital. Alternatively, advanced HIV infection might modulate the inflammatory response to viral infections in the CNS and, so, there is no pleocytosis. This might be relevant to the 6 of 39 patients with normal CSF and detectable EBV.

Our failure to detect HSV-2 and VZV in this cohort was unexpected, since both are common identifiable causes of viral meningitis in Western industrialised countries [16, 17]. It may be that the spectrum of causes of herpes viral meningitis in a population with high HIV prevalence is different to that in Western industrialised countries.

A striking finding was the high incidence of EBV in the CSF of HIV-infected individuals, even in those with a normal CSF. The presence of EBV in the CSF of people with HIV is usually associated with primary CNS lymphoma rather than meningitis [5, 18]. It is impossible to exclude the possibility that some of our patients may have had lymphoma, as brain imaging or biopsy were not routinely available at the time of investigation, but the clinical presentations of acute meningitis were very different to the chronic altered mental status or focal neurological deficit, which is usually seen in patients with CNS lymphoma [19].

The high detection of EBV in the CSF could be relevant to the clinical presentation or it could reflect activation in immunocompromised patients that is of no pathogenic significance. However, the detection of EBV was significantly higher in those with a raised CSF WCC. Ideally, we would have liked to explore the association of EBV with clinical outcome, but the retrospective nature of our study limited this. We recently described an association with high EBV titres in the CSF and poor outcome in patients with bacterial meningitis, which suggests that there might be more to this story [20].

In conclusion, we saw a different spectrum of herpes virus infections in this population than that seen in Western industrialised settings. EBV was frequently detected in the CSF, regardless of whether there was a pleocytosis or not, though higher viral titres were seen in patients with a pleocytosis and in HIV-positive patients. The significance of these findings merits further investigation.

## Abbreviations

HSV-1: Herpes simplex virus 1
HSV-2: Herpes simplex virus 2
VZV: Varicella zoster virus
EBV: Epstein–Barr virus
CMV: Cytomegalovirus
HIV: Human immunodeficiency virus
WCC: White cell count
RCC: Red cell count
CSF: Cerebrospinal fluid
CNS: Central nervous system
PCR: Polymerase chain reaction
